# A cell-level quality control workflow for high-throughput image analysis

**DOI:** 10.1186/s12859-020-03603-5

**Published:** 2020-07-02

**Authors:** Minhua Qiu, Bin Zhou, Frederick Lo, Steven Cook, Jason Chyba, Doug Quackenbush, Jason Matzen, Zhizhong Li, Puiying Annie Mak, Kaisheng Chen, Yingyao Zhou

**Affiliations:** 1 Genomics Institute of the Novartis Research Foundation, 10675 John Jay Hopkins Drive, San Diego, California 92121 USA; 2 Shiyu Children Foundation, Room 1006-1008, Genesis Beijing, No. 8 Xinyuan South Road, Chaoyang District, Beijing, 100027 PR China; 3 Janssen R&D LLC, 3210 Merryfield Row, San Diego, California 92121 USA

**Keywords:** Cell-level quality control, High throughput image analysis, Image quality measurement, Machine learning, CellProfiler

## Abstract

**Background:**

Image-based high throughput (HT) screening provides a rich source of information on dynamic cellular response to external perturbations. The large quantity of data generated necessitates computer-aided quality control (QC) methodologies to flag imaging and staining artifacts. Existing image- or patch-level QC methods require separate thresholds to be simultaneously tuned for each image quality metric used, and also struggle to distinguish between artifacts and valid cellular phenotypes. As a result, extensive time and effort must be spent on per-assay QC feature thresholding, and valid images and phenotypes may be discarded while image- and cell-level artifacts go undetected.

**Results:**

We present a novel cell-level QC workflow built on machine learning approaches for classifying artifacts from HT image data. First, a phenotype sampler based on unlabeled clustering collects a comprehensive subset of cellular phenotypes, requiring only the inspection of a handful of images per phenotype for validity. A set of one-class support vector machines are then trained on each biologically valid image phenotype, and used to classify individual objects in each image as valid cells or artifacts. We apply this workflow to two real-world large-scale HT image datasets and observe that the ratio of artifact to total object area (*AR*_*cell*_) provides a single robust assessment of image quality regardless of the underlying causes of quality issues. Gating on this single intuitive metric, partially contaminated images can be salvaged and highly contaminated images can be excluded before image-level phenotype summary, enabling a more reliable characterization of cellular response dynamics.

**Conclusions:**

Our cell-level QC workflow enables identification of artificial cells created not only by staining or imaging artifacts but also by the limitations of image segmentation algorithms. The single readout *AR*_*cell*_ that summaries the ratio of artifacts contained in each image can be used to reliably rank images by quality and more accurately determine QC cutoff thresholds. Machine learning-based cellular phenotype clustering and sampling reduces the amount of manual work required for training example collection. Our QC workflow automatically handles assay-specific phenotypic variations and generalizes to different HT image assays.

## Background

With the advance of high throughput (HT) imaging technology and related automated image analysis tools, scientists may conduct daily cell-level probes of biological systems under many treatment conditions [1–3]. However, as single cell data generation expands rapidly, e.g. in the field of cell profiling [4, 5], a critical challenge researchers face is automatically and reliably flagging images and cells contaminated by artifacts. Previous research has focused on studying and comparing metrics that detect image-level or patch-level artifacts such as blurring and saturated pixels [6, 7]. A widely accepted approach is to review these quality control (QC) metrics and identify per-assay, per-metric thresholds to accept or reject entire images or patches [8].

A fundamental limitation of this approach is that existing QC metrics not only reflect fluctuations in image quality but also shift with natural phenotypic variations. For example, DNA accumulation in apoptotic cells can lead to more saturated pixels, high protein expression ratios may lead the image intensity into a wider range, and a shift to fewer but larger cells leads to fewer edge pixels and therefore less high frequency components in the image power spectrum. The dependency of such QC metrics on cellular context such as cell counts and morphology can cause images with valid phenotypic variations to be discarded, leading to low true positive rates. Likewise, these QC metrics may also be unaffected by true cell-level artifacts, such as segmentation failures from treated cells adhering together and forming blobs, leading to low true negative rates as well.

The limitations of existing image-level QC metrics suggests the necessity of studying QC at the cell level. In addition to tackling the QC challenge in HT image analysis, cell-level artifact detection has also become increasingly vital due to the growing interest in studying and understanding the heterogeneous behavior embedded in cell subpopulations [9]. For samples composed of various subpopulations of cells, their diverse feature distributions can be skewed by the presence of bright foreign objects and poorly segmented cells. However, existing outlier detection methods that rely on the assumption of feature normality [8] are easily undermined because the morphological profile of a heterogeneous culture adopts diverse shapes. Model-based approaches provide an alternative solution, e.g., cell classifiers trained by providing normal or outlier examples [10–12]. Such data-driven strategies, however, require prior knowledge and ideally an exhaustive search for all possible phenotypes including real and artificial cells, which is rarely practical in the context of HT screening. For these reasons, a practical cell-level QC implementation remains a crucial unsolved problem.

To overcome the aforementioned limitations of current QC methods and improve the robustness of cellular heterogeneity studies, we developed a generalized (i.e., non-assay specific) QC workflow to flag individual artificial cells in HT imaging, enabling both more reliable cellular profiling and straightforward assessment of image quality as evaluated by the ratio of artifact area to total detected cell area (*AR*_*cell*_). To our knowledge, this represents the first QC method and scoring metric that operate at the cell level. Due to the lack of standardized ground truth image quality scores for HT image data to directly compare against, we demonstrate the benefits of our approach by applying it to two real-world assays, showing how it favorably compares to existing image QC methods in a variety of applications.

## Results

**Fig. 1:**
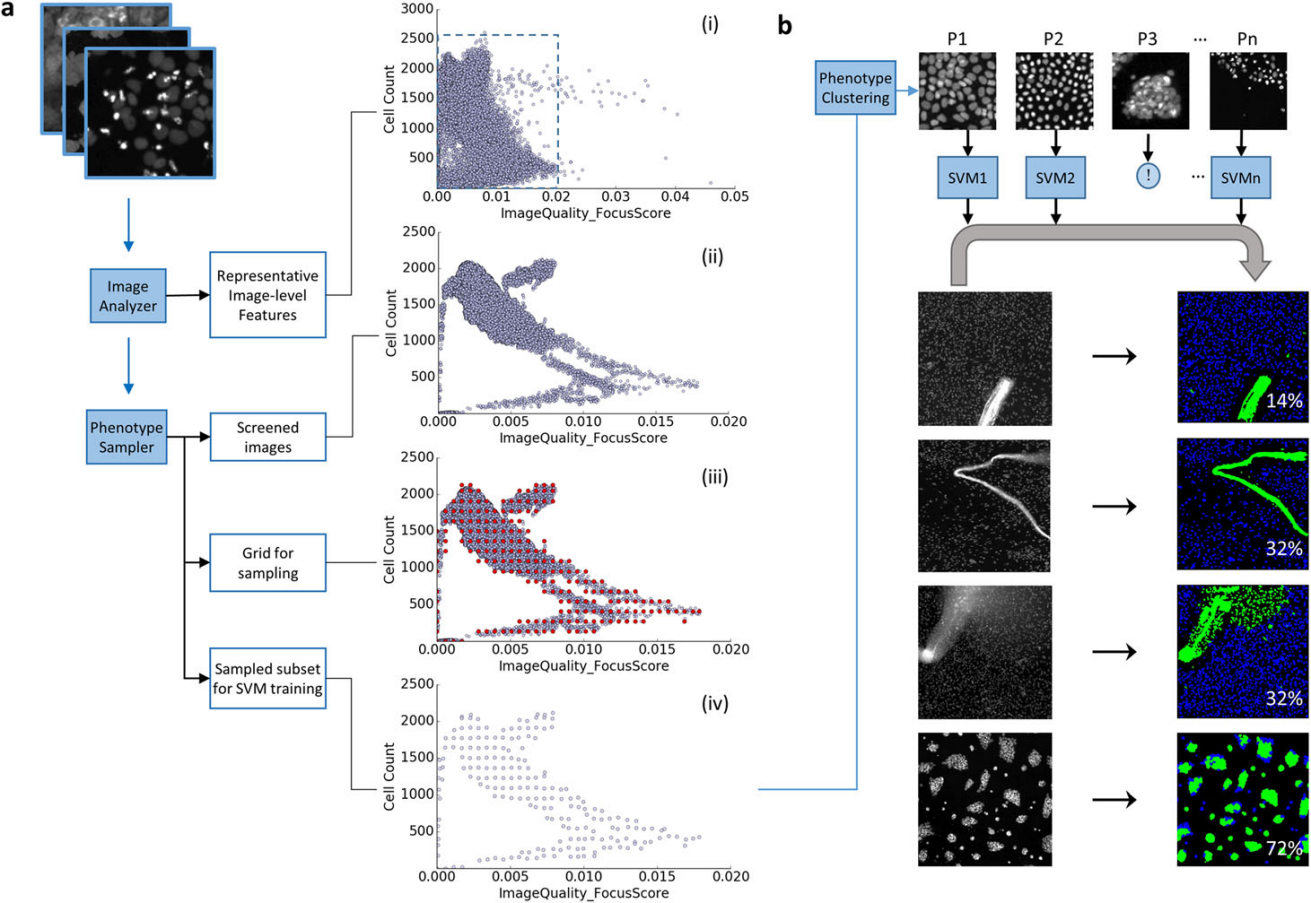
Schematic diagram of QC workflow. **a** A phenotype sampler to collect images that retain phenotypic variety. Grouped by their phenotypes, sampled images provide representative cells to train one one-class SVM per phenotype. (Dashed inset in (i) expanded in (ii-iv)). **b** A set of one-class SVMs to identify artifacts from each image. (‘!’ denotes a phenotype is excluded through visual inspection.) In all cell QC masks, artifacts are marked in green and good quality cells are labeled in blue, with the percentage of artifacts listed in white

Our cell-level QC workflow mainly consists of two parts, shown in Fig. 1. The first part (Fig. 1a) covers image analysis and feature selection to pick a representative but computationally feasible feature set, followed by phenotype sampling to collect an image subset that covers all phenotypes. The second part (Fig. 1b) builds a set of one-class support vector machines (SVMs), one per cellular phenotype, which then identify artifacts from each image of the assay. With the artifacts identified, *AR*_*cell*_ calculation is straightforward. In the following subsections we provide more details about our workflow.

### Image analysis and feature selection

We employ the open-source image analysis software CellProfiler ([2], http://www.cellprofiler.org/) to perform cell segmentation and quantitative measurements at both image and cell level using standard modules. As a result, every image is characterized by features including focus scores, intensity, correlation between pairs of staining markers (if more than one marker is collected), and signal-to-noise (SNR) ratio [13]. To minimize the dimensionality of the feature space while retaining phenotypic variety, we apply hierarchical clustering (implemented in scikit-learn, [14]) to identify groups of highly correlated image-level features. For both tested assays, the clustering reveals that feature ‘FocusScore’ is highly correlated with the standard deviation of pixel intensity (i.e., feature ‘StdIntensity’), while the other intensity measurements fall into one subgroup. We therefore select image features including ‘PowerLogLogSlope’, ‘FocusScore’, ‘MeanIntensity’, ‘Correlation’, and ‘SNR’ for the following QC process. Given that most image features are sensitive to the count of cells observed in the field of view [6], to minimize the impact of cellular phenotype fluctuation on QC performance, we also include ‘Cell Count’ in the final feature list for image sampling.

### Phenotype sampler

The premise of our workflow is to build a set of classifiers trained by a comprehensive collection of biologically valid phenotypes, and then identify an object as an artifact if it does not resemble any of these phenotypes. This new approach requires the collection of artifact-free cell samples, in order to treat artifact identification as an anomaly detection machine learning problem. To ensure the effective sampling of all artifact-free cells, our phenotype sampler takes two steps: outlier-based removal of artifact-dominated images, and phenotype-preserving image sampling.

First, we wish to identify and remove images that predominantly contain artifacts via image-level phenotype assortment (Fig. 1a.i-ii), since the biological phenotypes corresponding to these images are likely predominated by artifacts. We accomplish this with image-level outlier detection. Our outlier detection algorithm is based off our observation that HT screening images form clusters with variable densities in the multi-dimensional QC feature space (Fig. 1a.i, Supplementary Fig. 1) due to the nature of screening studies, which typically contain replicates of treatments, treatments at multiple dosages, and compounds sharing structures or targeting the same signaling pathways. Given that non-artifact images form clusters due to the aforementioned guilt-by-association forces, images falling outside clusters are suspect, likely corrupted by imaging, staining, or segmentation artifacts. We apply a density-based local outlier detector [15] to identify such out-of-cluster images while allowing for variation in the densities of the clusters themselves (Fig. 1a.ii).

Second, we subsample the remaining images to cover diverse phenotypes (Fig. 1a.iii-iv) and ensure equal representation of phenotypic variations in the sampled subset. This is particularly important for retaining rare but interesting phenotypes in the training collection. Our overall approach is to iteratively construct a sparse sampling grid in the full feature space from which we will sample the nearest images to build our training set. Sparsity is desirable because the number of possible feature-value combinations is very large and many regions of the feature space contain few or no images. We begin by creating an equidistant one-dimensional grid along each feature dimension at spacing decided by kernel density estimation (KDE, Fig. 1a.iii, [14]). Because image features extracted from HT imaging assays usually display various distributions, we apply KDE with 5-fold cross validation to obtain an optimal sampling bandwidth, *h*_*i*_, for the *i*th feature dimension and treat this *h*_*i*_ as a base sampling rate. The final sampling rate is determined by multiplying *h*_*i*_ with an integer *K*, which controls the tradeoff between sample set size and computation time. We iteratively construct the sparse, full-feature space sampling grid by starting with any two feature dimensions and, before adding another dimension, removing any grid point whose distance towards its best image match (a data point obtained through nearest neighbor search) is beyond *r* = $\sqrt{\sum_{i=1}^D{h}_i^2}$, where *D* is the dimensionality of the feature space in the current iteration. After iterating through all selected image features, the resulting multidimensional sampling grid is a low-resolution representation of the raw data set (Fig. 1a.iii). Images are then sampled by matching these grid points with their nearest neighbors (Fig. 1a.iv).

### Single cell classifier for QC

With our phenotype-preserving sample set created, the remainder of our pipeline groups related images into phenotype-representing clusters, removes clusters corresponding to artificial cells, trains one-class SVM classifiers, and finally labels single segmented cells as artifacts or not (Fig. 1b). For phenotype grouping, we first apply a Gaussian mixture model (Python Scikit-learn library [14];) to estimate the number of groups, *n*_up_, using Bayesian information criterion (BIC). To handle the variation in feature distribution with respect to different subgroups and choose a more suitable number of effective subgroups automatically, sampled images are then clustered by a variational Bayesian Gaussian Mixture model [14] with the number of groups upper-bounded by *n*_up_. With the grouping determined, we output a set of representative images from each group for manual inspection to determine whether the group represents artifacts. Artifacts may be biologically valid but algorithmically problematic, for example, treated cells may adhere to each other and form blobs that cannot be segmented (Fig. 1b, phenotype 3). This manual inspection step provides an opportunity to catch any remaining quality issues at a much lower time cost than upfront analysis as only a small number of images per group need to be checked. It is important to our overall method that artifact phenotypes be flagged here. In our later discussion of both assays we apply our method to we will demonstrate that flagging artifact phenotypes is simple for users. From each distinct phenotype group, cells are collected and screened by a cell-level density based outlier detector [15] to achieve a tighter group envelope before training a one-class SVM classifier with radial basis function (RBF) kernel [14] to classify objects belonging to that phenotype. After training the classifiers to learn the decision boundary that envelops the feature space occupied by the representative cells, a candidate cell is recognized as an artifact upon unanimous agreement by all SVMs (Fig. 1b), i.e., an object is labelled an artifact if it is located outside all the boundaries of normal cell groups represented by those SVMs. The ratio of artifact area to total segmented object area, *AR*_*cell*_, is output as a direct measurement of image quality (Fig. 1b).

Each one-class SVM is defined by two parameters: *nu* controls the fraction of training errors and *gamma* shapes the RBF kernel. We use the default setting (one over the number of cell descriptors) for *gamma*. We choose a small constant for *nu* (1E-3) since we have high confidence in the sampled inlier training data output by our processing pipeline and want the SVM to positively identify a very high proportion of this data. The impact of different choices for parameters *nu* and *gamma* on the *AR*_*cell*_ metric will be demonstrated later with a real-world case study.

An example histogram of *AR*_*cell*_ values and corresponding image masks is given in Supplementary Fig. 2. We recommend examining the *AR*_*cell*_ histogram to gain intuition about the image quality distribution. In the given example, due to the long tail of the distribution, a stringent, low *AR*_*cell*_ threshold could be set without sacrificing a large percentage of images. Alternatively, an intuitive absolute threshold such as 50% could be used if the downstream processing pipeline is more robust against whatever anomalies are observed in the higher-scoring images. A QC process necessitates making a tradeoff between saving partially contaminated images and increasing statistical confidence, and this choice is intrinsic to the nature and goals of each study and assay. This approach helps quantify the decision-making process while still allowing room for domain-specific decision-making by the users.

### Phenotype sampling and cell segmentation

Our approach targets artifacts at the cell level, relying on a preprocessing stage (i.e. CellProfiler) for segmenting the cells. This segmentation in turn is usually limited by the number of images or phenotypes tested during the development of the preprocessing image segmentation solution. Thus, in our framework, we also apply the phenotype sampler ahead of designing the preprocessing cell segmentation solution (Supplementary Fig. 2), ensuring the appraisal of diverse phenotypes to maximize segmentation quality.

In the event of errors in this preprocessing segmentation, clumps may be improperly segmented or not segmented at all. Our method is robust against such failures in two ways. First, if the number of images featuring improperly segmented artifacts is small, it is unlikely that they will pass the outlier detection stage. If the segmentation-based artifacts are instead of a sufficient density to pass the outlier detection, they will manifest as one or more phenotype clusters during the phenotype detection stage. The user will have an opportunity to review the clusters and decide to allow the segmentation artifacts as their own phenotype or deny them as artifacts. Which choice is more desirable depends on the specific goals of the study and how much useful information can be extracted from these problematic cases.

### Cell-level QC performance of assay α

**Fig. 2:**
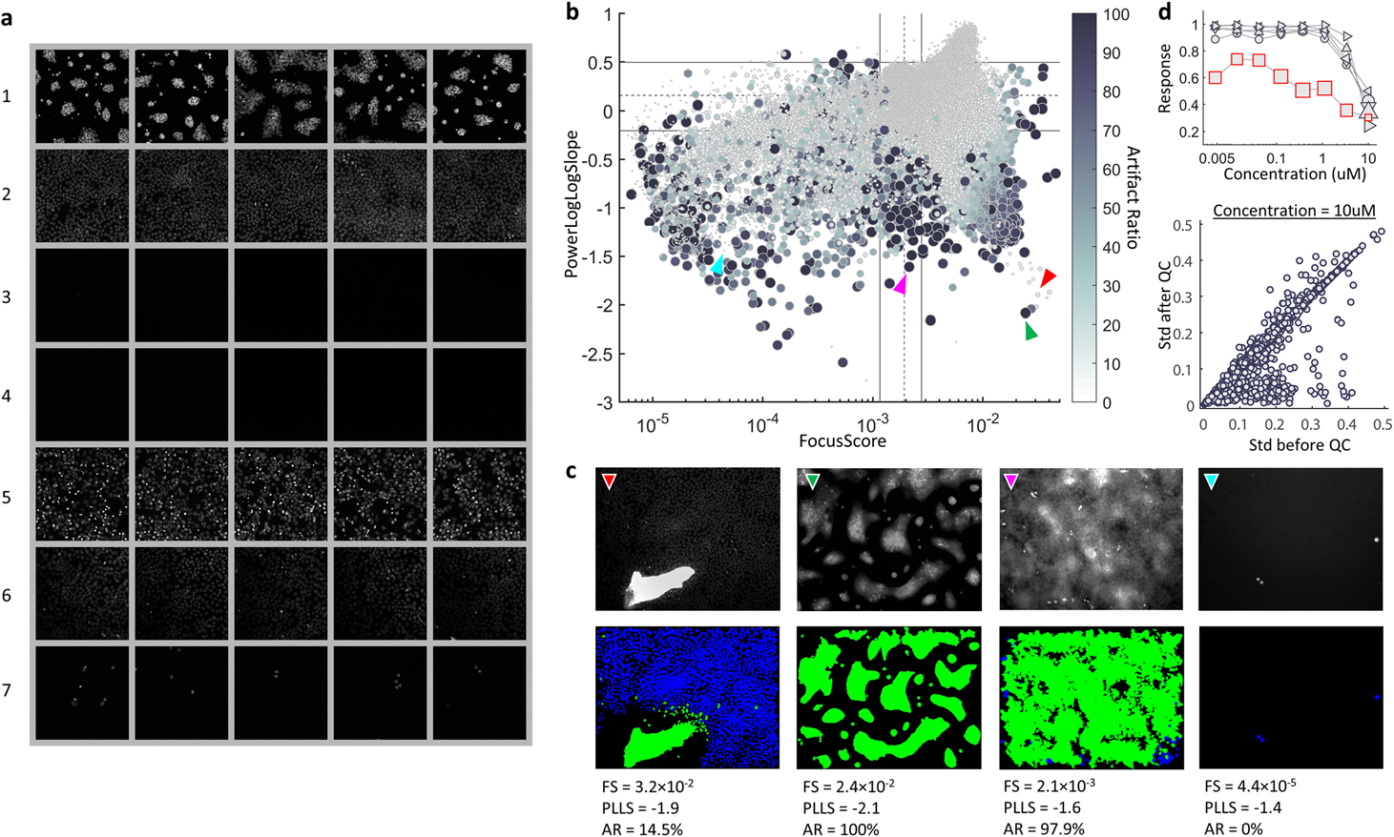
Performance of cell-level QC, assay α. **a** Images sampled by our phenotype sampler are grouped into 7 phenotypes with 5 examples shown for each. **b**-**c** A comparison between conventional image quality measurements and *AR*_*cell*_. Each dot represents one image with the size and intensity proportional to *AR*_*cell*_. For the conventional QC metrics, FocusScore (FS) and PowerLogLogSlope (PLLS), the dashed line indicates the median value and the solid lines show 1st quartile - IQR*1.5 and 3rd quartile + IQR*1.5 respectively (IQR = interquartile range). Examples of cell-level QC result (colored arrows) are listed with their FS, PLLS and *AR*_*cell*_ (AR) in (C). In all QC masks, artifacts are marked in green and good quality cells are labeled in blue. **d** Cell-level QC improves the accuracy of well-level phenotype summary. Top, an example of inconsistent dose response: out of six replicates (grey curves), the outlier curve (marked by red squares) contains images with *AR*_*cell*_ between 3.8 and 63.0% as indicated by marker size. Bottom, comparison of drug response consistency before and after cell-level QC at concentration equal to 10uM (see Supplementary Fig. 7 for other concentrations). Each circle represents one compound

We first evaluated the performance of our cell-level QC workflow using HT image assay α. Our phenotype sampler collected 3108 images primarily encompassing seven image-level phenotypes (Fig. 2). Next we manually inspected the phenotype sample images. In image phenotype 1, cells mostly clump together and cannot be segmented properly through CellProfiler, therefore that phenotype was considered an algorithmic artifact and thus excluded from subsequent one-class SVM training. Phenotypes 2, 6, and 7 contain cells that represent the dominant cell population, differentiated by an uneven illumination that dims towards the bottom left of the field (phenotype 6), and by lower seeding density cultures (phenotype 7). Phenotypes 3 and 4 mostly contain empty wells with different background noise levels. Phenotype 5 contains a high apoptosis ratio, shown by the occurrence of bright spots. After collecting cells from the desired phenotypes [2, 5–7] and training the one-class SVMs, every detected object in assay α was classified as a valid cell or artifact and *AR*_*cell*_ was calculated to summarize each image’s quality.

We then compared the result with conventional image QC measures, e.g. FocusScore (FS) and PowerLogLogSlope (PLLS) (Fig. 2, Supplementary Fig. 4) and found that *AR*_*cell*_ was more practical than the other QC measures in ranking images by quality since it was less sensitive to cell count fluctuations. As a single tuning parameter, *AR*_*cell*_ was more practical and accurate in ranking image quality, e.g., two images receiving similar FS and PLLS score but with one clearly less contaminated could be ranked more accurately by *AR*_*cell*_ (Fig. 2 b-c, red vs. green arrow). Unlike with *AR*_*cell*_, we could not reliably rank image quality using any single conventional quality measure, as an image sitting at the normal range of one measure could be identified as an outlier by another (e.g., Fig. 2 b-c, magenta example). As *AR*_*cell*_ is calculated at cellular granularity, it performs accurately under a wide variety of image cell counts when compared to conventional quality metrics. For example, among all four examples listed in Fig. 2c, the cyan one obtained the lowest FS as a result of low cell count, but its quality measurement was justified by *AR*_*cell*_. Third, thresholding the conventional QC measurements is nontrivial due to the complex distributions of quality metrics driven by cellular phenotype fluctuation (Fig. 2b) as well as undesired technical variations. Gating on additional QC measurements requires accurately setting additional thresholds for each added feature to ensure identification of imaging and staining artifacts.

In Supplementary Fig. 5 we experimented with different plausible error rates for *nu* and *gamma* when training the SVMs for this assay. Part A shows the *AR*_*cell*_ values and corresponding image masks (artifacts in green, valid cells in blue) for an artifact-containing image under different parameter values. Higher *nu* and *gamma* tighten the decision boundary of the SVM classifier around the cluster of training images corresponding to a particular phenotype, while lower *nu* and *gamma* expand the boundary to be more permissive. In Part B we find that varying our chosen value of *nu* = 1E-3 by a factor of 10 changes *AR*_*cell*_ by over 1% in 0.08% of images (at 1E-4) and 6.3% of images (at 1E-2). Since the image clusters have higher density towards the center of each cluster, we expect this behavior whereby increasing *nu* has a larger effect on *AR*_*cell*_ than decreasing it. While the parameter values we list provided accurate classification for both of our assays, users can replicate this parameter variation experiment if they find the SVM classifiers are too permissive or restrictive.

Observing the prevalence of heterogeneity in assay α (Supplementary Fig. 6A), we scored well-level response by the fraction of cells having the protein of interest translocated into the nucleus. We investigated the impact of artifacts on this measurement and found that removal of artificial cells restored the distribution of population heterogeneity (Supplementary Fig. 6). For this assay, among six replicates, distortion in dose response as a result of artificial cells was observed for some compounds (e.g., Fig. 2d, top). We then compared the consistency of dose response measurements before and after cell-level QC by calculating the standard deviation of replicates collected for each compound at multiple doses. After removing artificial cells, we recalculated well-level responses and excluded wells predominated by artifacts (i.e., *AR*_*cell*_ > 50%), as the remaining non-artifact cell population may not be sufficient to characterize the well-level phenotype (e.g., Fig. 2c, magenta example). As indicated by the standard deviation of replicates, improvement was observed in response consistency across all concentrations (Fig. 2d, bottom, Supplementary Fig. 7).

### Cell-level QC performance of assay β

**Fig. 3:**
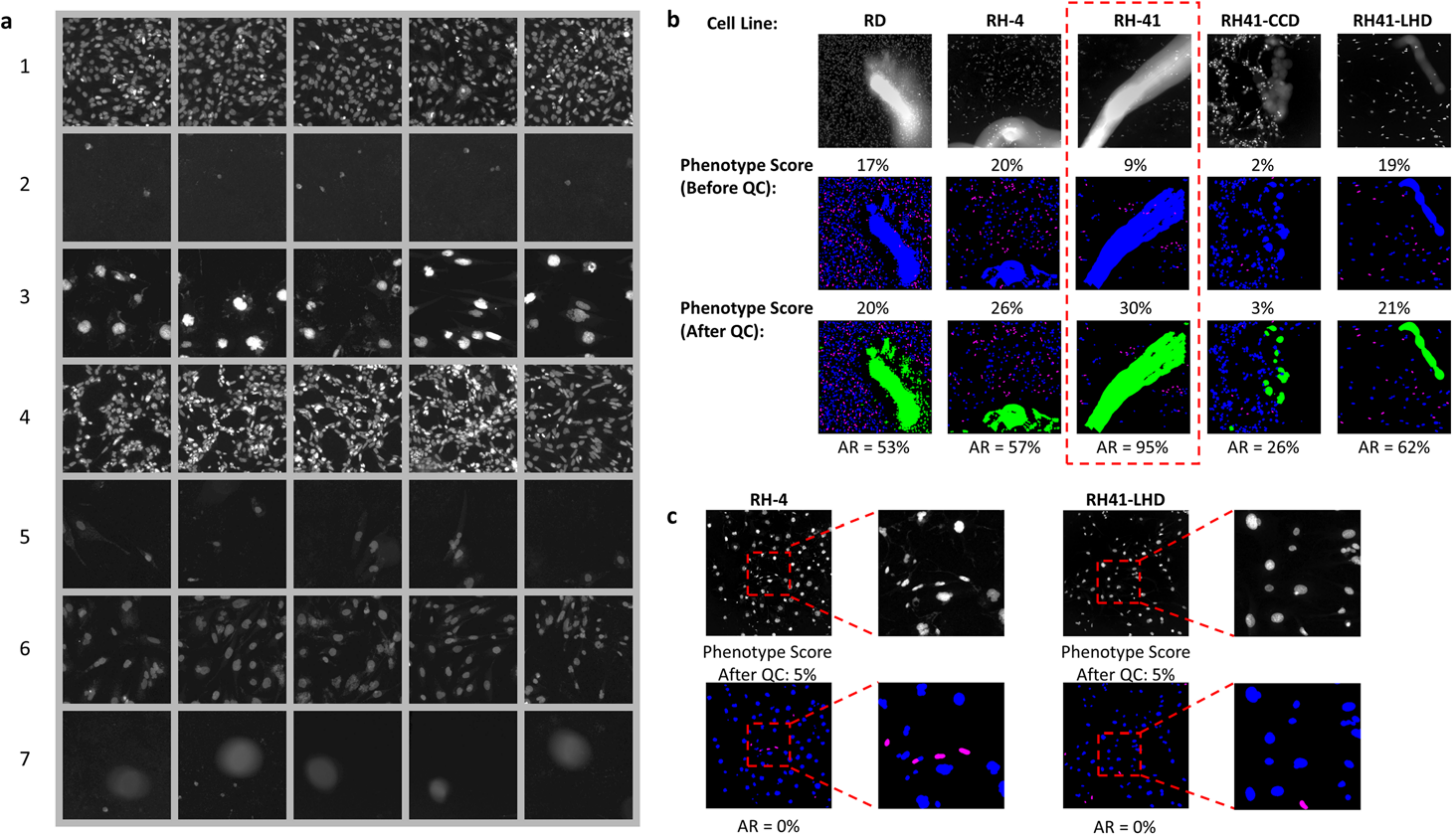
Performance of cell-level QC, assay β. **a** Examples of sampled images grouped by their phenotypes. Sampled images were grouped into seven phenotypes with five examples shown for each. For visualization purposes, only a representative region of the original well image is displayed. **b** A comparison of phenotype score before and after cell-level QC for different cell lines. From top to bottom are examples of raw images and their phenotype masks before and after QC. Red dash line box shows a well dominated by artifacts (*AR*_*cell*_ > 70%) and removed before downstream analysis. **c** Examples of wells containing heterogeneous subpopulations to show minority phenotypes (e.g., cells labeled in magenta) were not mistakenly labeled as artifacts by our cell-level QC approach. In all masks, nuclei with phenotype of interest are labeled in magenta, otherwise blue; detected artifacts are marked in green

We then evaluated this QC workflow on a second, significantly more complex assay β, collected by a different imaging system for scoring a morphologic phenotype of interest for multiple cell lines: small and elongated nuclei. After image-level feature extraction and cell segmentation, 1734 images were sampled and grouped into seven phenotypes (Fig. 3a). During the manual inspection phase of sample images from each of these seven image phenotypes, image phenotype seven was removed because it exhibited a common staining artifact (Fig. 3a, bottom row). The grouping result shows that assorted phenotypes regarding cellular morphology, intensity, spatial distribution and culture density are captured by our phenotype sampler. Because of the relatively low seeding density of this assay (~ 700 cells/well), staining artifacts could easily dominate an image. Therefore, for this assay, we raised the threshold of *AR*_*cell*_ to 70% to eliminate very problematic well images and preserve less contaminated ones (Fig. 3b). Following cell-level QC, we recalculated the phenotype score, i.e., the ratio of small elongated nuclei contained in a well, and obtained a more accurate description of well-level phenotype regardless of the cell line (Fig. 3b). Importantly, our method was not affected by the heterogeneity in cellular populations and retained minority phenotypes in heterogeneous cultures (Fig. 3c).

### Comparison with a patch-level QC approach

**Fig. 4:**
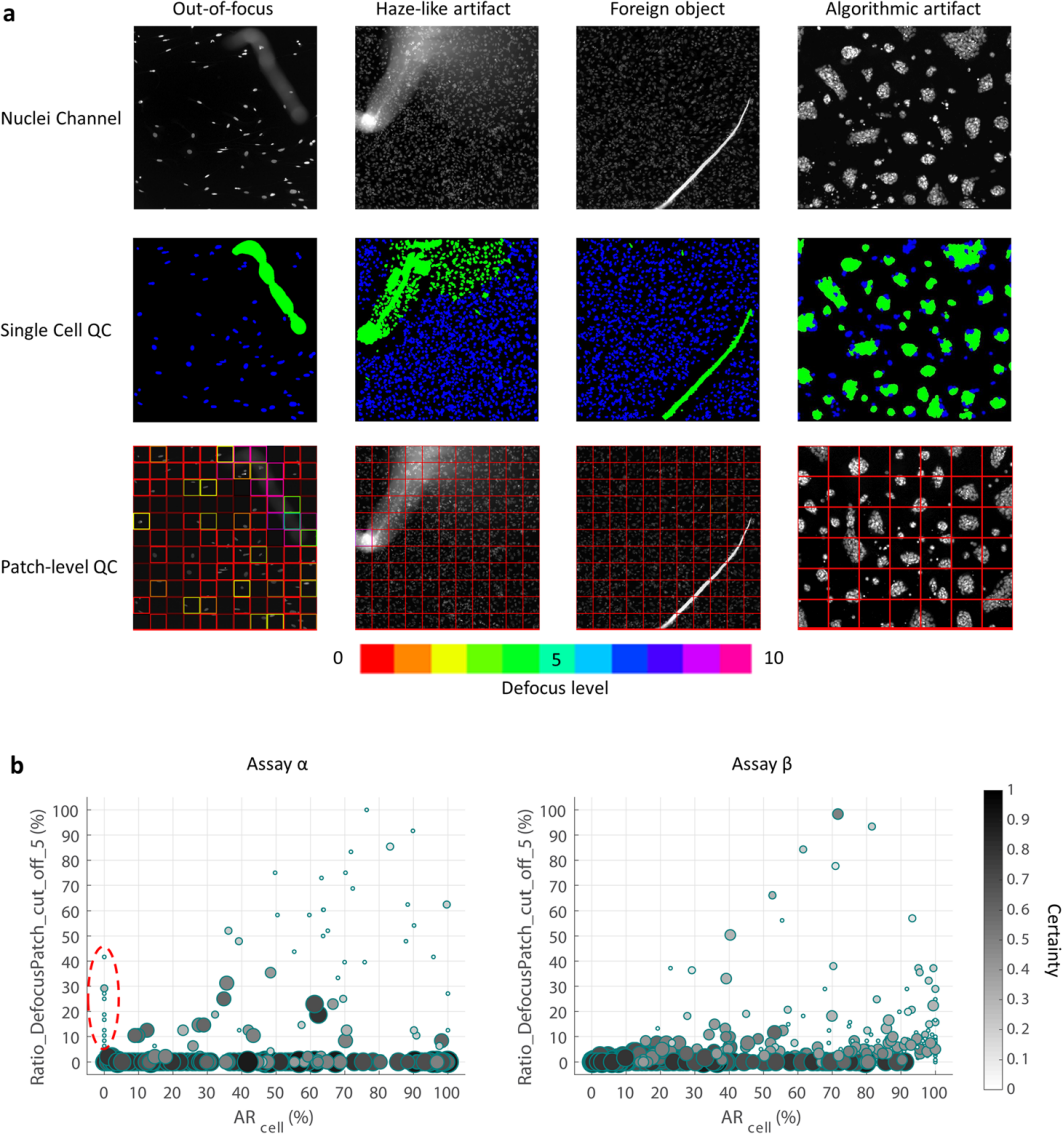
Detection of staining artifacts, a comparison between our cell-level QC and a patch-level QC application recently integrated into common image analysis platforms. **a** From left to right, four different types of artifacts are displayed with their: top - raw image, middle - single cell QC result, and bottom - patch-level defocus score generated by a deep learning approach (Yang 2018). In the cell QC mask, artifacts are marked in green and good quality cells are labeled in blue. In the results generated by the patch-level approach, the patch outlines denote the predicted defocus level by hue (red for least defocus) and prediction certainty by lightness (increased lightness for increased certainty). **b** A comparison between the ratio of defocused patches (y-axis) and *AR*_*cell*_ (x-axis) for images collected from assay α and β. Each dot represents an image with both the size and intensity proportional to the prediction certainty of defocus level. Patches with defocus level greater than 5 are considered out of focus. Images within the red dashed circle show high defocus patch ratio but low prediction certainty. More comparisons using different defocus level cutoffs can be found in Supplementary Fig. 8 along with labeled examples

In our final experiment, we compared the performance of our cell-level QC workflow with a recent patch-level image QC tool integrated with ImageJ and CellProfiler ([7], Fig. 4). Four different types of artifacts including out-of-focus, haze-like, foreign object or saturation signal, and algorithmic (segmentation failure) were tested. Both methods were able to locate out-of-focus regions but at different resolutions: cell- versus patch-level. However, the patch-level tool was unable to recognize those artifacts not involving focus issues. Our cell-level QC workflow covered more artifact varieties and successfully located them, including the one artificially created by an image segmentation failure (Fig. 4a). We further summarized the patch-level QC result by calculating the ratio of patches with defocus level greater than a fixed threshold (Fig. 4b, Supplementary Fig. 8A) and comparing it against *AR*_*cell*_. A high *AR*_*cell*_ is correlated with a high defocus patch ratio, except for those images containing mostly background pixels for which the patch-level QC tool reports very low certainty (Fig. 4b, red dashed circle). Reducing the defocus level cutoff permits patches with minor defocus issues, and recruits more patches with lower certainty from our tested images (Supplementary Fig. 8). Also, as expected, images with a high *AR*_*cell*_ obtained a low defocus patch ratio if they contained other in-focus artifacts (Fig. 4b and Supplementary Fig. 8).

## Discussion

We have presented a QC workflow built on two novel insights that gives users more robust and fine-grained quality control capabilities, while avoiding the lengthy and difficult process of simultaneously thresholding multiple QC features for every assay. The first insight is that cell-level artifact detection enables a single powerful, phenotype-invariant, image-level QC measure in the form of *AR*_*cell*_, the ratio of artifact to total object area in an image. The second insight is that high-quality cell-level artifact detection can be achieved through machine learning techniques: the properties of screening assays permit an unsupervised clustering approach to detect valid phenotypes with greatly lessened user intervention, and these phenotype clusters can then be sampled to train a set of classifiers to perform the aforementioned cell-level artifact detection.

Our results demonstrate the utility of model-based classification in filtering out artifacts while retaining valid phenotypes across the feature space of image quality metrics. Model-based classification approaches require manual sampling of training examples, making it extremely likely to overlook phenotypes of biological interest within the hundreds of thousands of well images in a production HT imaging assay. Our workflow avoids this issue and saves user effort by performing phenotype grouping using an unlabeled clustering methodology, built upon assumptions that are sound in the context of HT screening. It takes advantage of the guilt-by-association property embedded in HT screening whereby multiple images share biologically relevant phenotypes, and so implements a density-based outlier detector to remove images with prominent artifacts before sampling. To eliminate any remaining non-outlier artifacts from training examples, we group sampled images by their phenotypes for ease of inspection.

We demonstrated the capabilities of our proposed QC workflow by applying it to two large-scale HT screening assays, covering a range of cell lines, treatments, and image acquisition settings. Our QC metric *AR*_*cell*_ provides an intuitive quality ranking more in line with human visual assessment than previous scoring systems. Gating on *AR*_*cell*_, images largely contaminated by artifacts can be excluded and less impacted ones can be salvaged before downstream analysis. In our experiments, we successfully remove artifact noise with our cell-level QC tool, enabling more accurate subpopulation identification and precluding the need for noise-reducing aggregation approaches that discard meaningful biological information.

Our QC workflow identifies artifacts at cell level, using an external cell segmentation solution. The phenotype grouping and manual inspection of phenotype sample images acts as a last line of defense against common artifacts, but on rare occasions erroneously segmented fragments from large artifacts may resemble valid phenotypes closely enough to be misclassified (Supplementary Fig. 8B). Therefore it is preferable whenever possible to improve the segmentation itself so that more of an assay’s cells can be used for analysis instead of discarded. To help achieve reliable cell segmentation results, our phenotype sampler component is run independently as part of preprocessing to contribute a collection of phenotype images to aid segmentation algorithm tuning. As emerging technologies drive the improvement of image segmentation methods, e.g., deep learning-based models [16, 17] we expect further advances in the performance of our proposed QC workflow by further reducing the risk of segmentation artifacts resembling valid cells passing through. Another direction for further study involves further reducing or eliminating the human analysis step via automated classification of image phenotypes as biologically valid or invalid before SVM training. Finally, testing on additional image datasets will enable a more comprehensive evaluation of workflow performance and facilitate tuning of model parameters for further generalization, leading to application of our workflow on more HT image assays including additional cell types, organoids, phenotypes, and 3D images.

## Conclusions

Image QC plays a very important role in image-based HT screening as it determines the quality of any downstream analysis. Our image QC workflow increases the resolution of QC analysis beyond existing image- or patch-level approaches to the cellular scale, robustly identifying previously undetectable artifacts. Our workflow automatically handles assay-specific phenotypic variations by collecting representative cells capturing an exhaustive range of non-artifact phenotypes from sampled images, identifying artifacts by comparing each candidate cell with the training examples, and detecting artificial cells created not only by imaging or staining artifacts but also the limitations of image segmentation algorithms. As a consequence, cells and images that were falsely accepted or rejected by existing methods are now properly classified, increasing the accuracy and sample size of all downstream analysis.

## Methods

The datasets used in the experiments were two HT image assays, α and β, previously collected in-house. Assay α is designed for scoring protein translocation in cells treated by compounds at multiple doses (see Supplementary Methods [18]). This assay contains 555,196 well images acquired on the PerkinElmer Opera QEHS system with six replicates for each condition. The average number of cells per image is ~ 1700. The second assay, β, is collected by a different imaging system (ImageXpress Micro High-Content Imaging System) for scoring a morphologic phenotype of interest (see Supplementary Methods) with seeding density of ~ 700 cells per well. Due to the use of multiple cell lines in assay β, we observe a larger variety of cellular phenotypes regarding morphology and staining intensity from its 230,382 wells.

## Supplementary information

**Additional file 1: Section 1.** Sample preparation and imaging. **Figure S1.** Images from a HT assay form clusters in the multi-dimensional feature space, with two such feature dimensions plotted. **Figure S2.** Example application of *AR*_*cell*_ for QC inspection, ranking, and thresholding. **Figure S3.** Representative images are collected to build a robust cell segmentation solution. **Figure S4.** A comparison between image-level QC measures and *AR*_*cell*_ in Assay α. **Figure S5.** Effect of one-class SVM hyperparameter *nu* and *gamma* variation on well image *AR*_*cell*_ scores. **Figure S6.** Cell-level QC improves the accuracy of population analysis. **Figure S7.** Dose response consistency across replicates before and after cell-level QC, assay α. **Figure S8.** A comparison between *AR*_*cell*_ and defocused patch ratio under different defocus level cutoffs.

## Data Availability

A python implementation of this cell-level QC workflow is available from our website https://carrier.gnf.org/publications/ImageQC_BMCBioinformatics/, where we also provide a representative subset of the datasets generated and analyzed during the current study to demonstrate the usage of our cell-level QC workflow.

## Abbreviations

QC: Quality control
AR: Artifact ratio
SVM: Support vector machine
SNR: Signal-to-noise ratio
KDE: Kernel density estimation
BIC: Bayesian information criterion
RBF: Radial basis function
PLLS: Power log-log slope
FS: Focus score
HT: High throughput
