# Supplementary material for: A cell-level quality control workflow for high-throughput image analysis
Source: BMC Bioinformatics. 2020 Jul 2;21:280. doi: 10.1186/s12859-020-03603-5 (PMC7333376; doi:10.1186/s12859-020-03603-5)
Supplement: Supplementary file 1 — Additional file 1: Section 1. Sample preparation and imaging. Figure S1. Images from a HT assay form clusters in the multi-dimensional feature space, with two such feature dimensions plotted. Figure S2. Example application of ARcell for QC inspection, ranking, and thresholding. Figure S3. Representative images are collected to build a robust cell segmentation solution. Figure S4. A comparison between image-level QC measures and ARcell in Assay α. Figure S5. Effect of one-class SVM hyperparameter nu and gamma variation on well image ARcell scores. Figure S6. Cell-level QC improves the accuracy of population analysis. Figure S7. Dose response consistency across replicates before and after cell-level QC, assay α. Figure S8. A comparison between ARcell and defocused patch ratio under different defocus level cutoffs. [file 12859_2020_3603_MOESM1_ESM.docx]

**Supplementary Methods**

**Sample preparation and imaging.** *Assay α: YAP translocation in HaCaT cell*. HaCaT cells (Boukamp, 1988) were cultured in DMEM containing 100 units/ml penicillin, 100 ug/ml streptomycin, 292ug/ml L-glutamine (Life Technologies, Inc), and 10% FBS (HyClone, SH30071.03) at 37°, 5% CO_2_, and 100% humidity. Cells were grown in T175 flasks, detached by trypsin, counted by Vi-Cell XR Cell Viability Analyzer, and seeded into 1536-well clear bottom assay plates (Greiner Bio-One, custom) at a density of 5500 cells/well in 5ul media by the GNF Systems Washer/Dispenser. After 24h at 37°, 8 point half-log serially diluted compounds were added using Pintool (GNF Systems), yielding a final dose response concentration range of 4nM-10uM. Plates were covered using GNF custom assay plate lids and incubated at 37° for 24h. The GNF Systems Washer/Dispenser was then used to perform all fixation and staining steps. The HaCaT cells were fixed by dispensing 2.5ul PFA (EMS, 15714-S) to each well for 4% final PFA at room temperature for 45m. The wells were aspirated leaving 2.5ul residual volume per well, then permeabilized and blocked by dispensing 2.5ul 0.2% Triton X100 (Sigma, T8787) and 3% BSA (Sigma, A7284) in DPBS (HyClone, SH30028.03) for 30m at room temperature. The wells were then aspirated leaving 2.5ul residual volume before dispensing 2.5ul 1:500 anti YAP 63.7 primary antibody (Santa Cruz Biotechnology sc-101199), 1:1000 Draq5 nuclear stain (Biostatus, DR50050), and 1.5% BSA in DPBS at room temperature for 4h. Next, the wells were washed with DPBS before dispensing 2.5ul 1:500 AF546 Goat anti-mouse IgG (Invitrogen, A11030) and 3% BSA in DPBS at room temperature for 4h. After another and final wash with PBS, the plates were sealed using an automated heat sealer. Images were acquired on the PerkinElmer Opera QEHS. One field of view per well was acquired using a 10X objective, 200 ms exposure times, and 540/75 and 690/70 emission filters with 2 X 2 binning.

*Assay β: Morphological profile of RD and RH4 cells.* RD, RH4 and RH4 derivative cells were cultured in DMEM containing 100 units/ml penicillin, 100 ug/ml streptomycin (Life Technologies, Inc), and 10% FBS (HyClone, SH30071.03) at 37°, 5% CO_2_, and 100% humidity. Cells were grown in T175 flasks, detached by trypsin, counted by Vi-Cell XR Cell Viability Analyzer, and seeded into 1536-well clear bottom assay plates (Greiner Bio-One, custom) at a density of 700 cells/well in 7ul media by the GNF Systems Washer/Dispenser. After 24 h at 37°, compounds were added using Echo 555 Liquid Handler (Labcyte) and the plates were covered using GNF custom assay plate lids and incubated at 37° for 96h. The GNF Systems Washer/Dispenser was then used to perform all fixation and staining steps. The RD and RH4 cells were fixed by dispensing 2.5ul PFA (EMS, 15714-S) to each well for 4% final PFA at room temperature for 30m. The wells were aspirated leaving 2.5ul residual volume per well, then permeabilized and blocked by dispensing 2.5ul 0.4% Triton (Sigma, T8787) with 3% BSA (Sigma, A7284) in DPBS (HyClone, SH30028.03) for 1h at room temperature. The wells were then aspirated leaving 2.5ul residual volume before dispensing 2.5ul 1:250 MF20 primary antibody (eBioscience, 14-6503) in DPBS with 1.5% BSA at room temperature for 4h. Thereafter, the wells were washed with DPBS before dispensing 2.5ul of 1:500 goat anti-mouse Alexa Fluor 488 (Life Technologies, A11029), 1:500 Hoechst 33342 (Life Technologies, H3570), 1:250 Alexa Fluor 647 Phalloidin (Life Technologies, A22287), 0.2% Triton X100, and 3% BSA in DPBS at room temperature for 4h. After another wash with DPBS, the plates were sealed using an automated heat sealer. One image was acquired per well on the ImageXpress Micro High-Content Imaging System (Molecular Devices).

**Supplementary Figures**


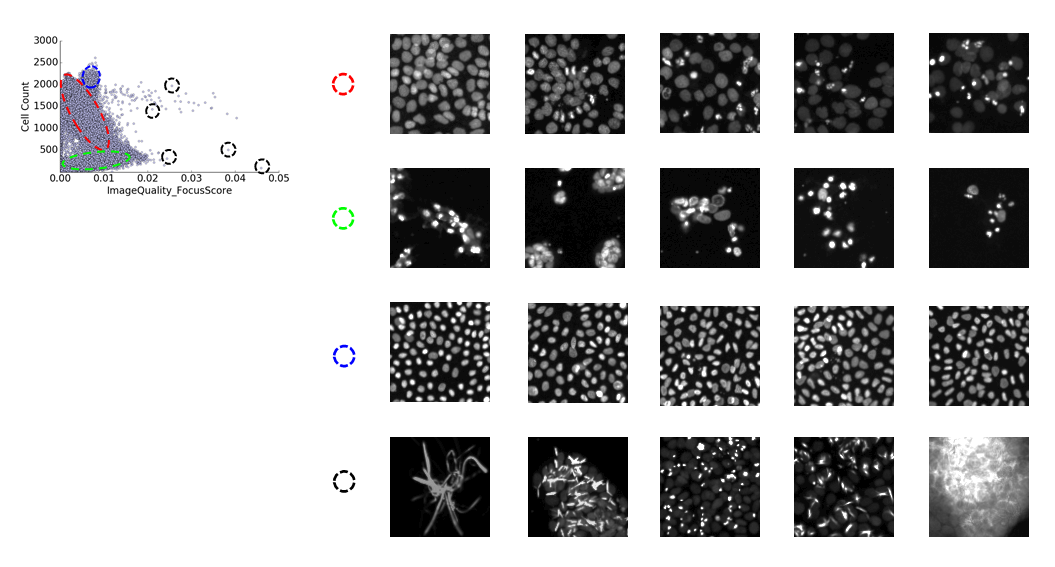


**Figure S1: Images (gray dots) from a HT assay form clusters in the multi-dimensional feature space, with two such feature dimensions plotted.** Images within the red, green, and blue groups share biological phenotypes. Black circles mark images likely containing prominent artifacts that will be removed by a density-based outlier detector (Fig. 1A.ii). Cells present phenotypic variation across each phenotype group’s images. This variation is preserved by our phenotype sampler (Fig. 1A).


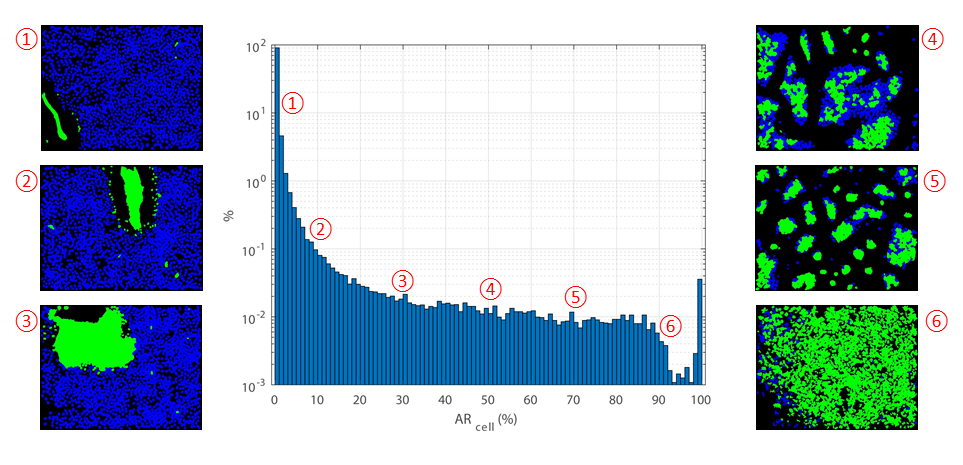


**Figure S2: Example application of *AR_cell_* for QC inspection, ranking, and thresholding.** Center: histogram of *AR_cell_* values for the well images in an assay. Sides: QC result masks sampled from various histogram bins for illustration (detected artifacts in green). The *AR_cell_* histogram provides quantitative feedback regarding the *AR_cell_* distribution, while visual inspection of images along the distribution can reveal specific patterns or types of artifacts that may influence choice of threshold or alert users to systemic issues.


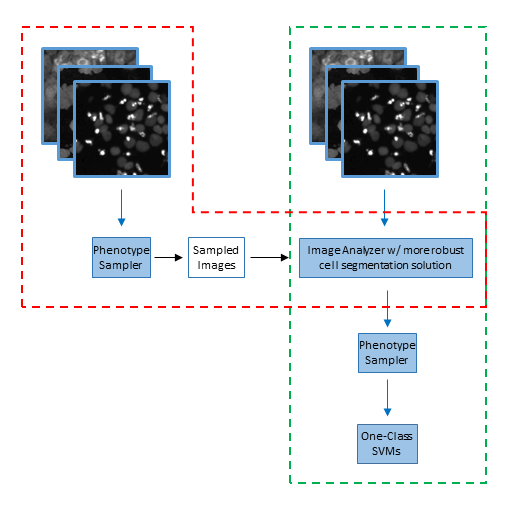


**Figure S3: Representative images are collected to build a robust cell segmentation solution.** To provide a more robust cell segmentation solution for our QC workflow (green dashed line box), representative images are collected by our phenotype sampler and used to test the image analysis solution (red dashed line box) so that a more accurate cell count and outline can be obtained for each phenotype.


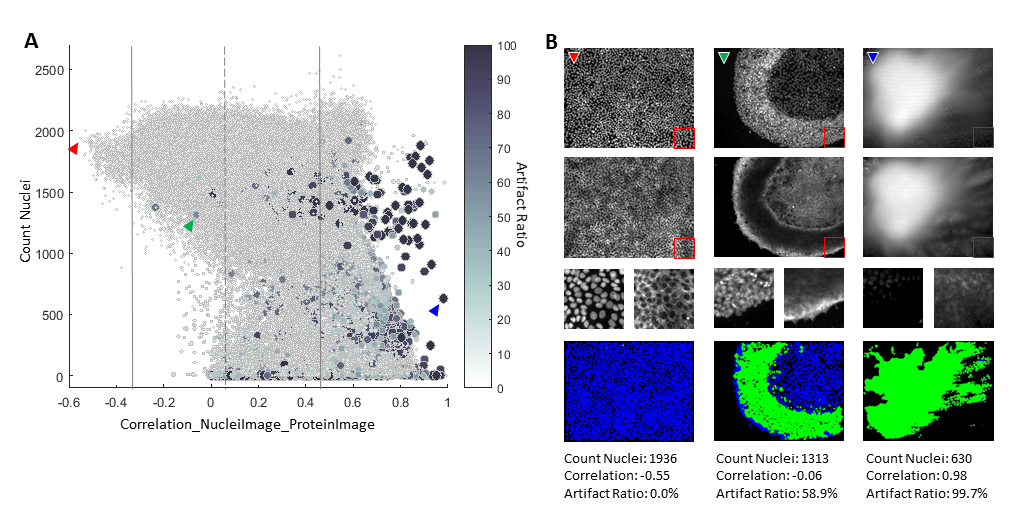
 **Figure S4: A comparison between image-level QC measures and *AR_cell_* in Assay α.** (A) Each dot represents an image with both the size and intensity proportional to *AR_cell_*. Feature ‘Correlation_NucleiImage_ProteinImage’ (henceforth abbreviated ‘Correlation’) calculates the correlation between the nucleus and the second protein marker channel. The dashed line shows the median and solid lines show 1^st^ quartile - IQR*1.5 and 3^rd^ quartile + IQR*1.5 respectively (IQR = interquartile range). (B) Three examples as pointed by arrows in (A) are listed. In each column, from top to bottom: nuclei channel, protein marker channel, enlarged regions from red boxes (with left tile for nuclei channel and right for protein marker), cell-level QC result mask (artifacts in green, valid cells in blue), coordinates from (A), and *AR_cell_*. Red arrow – an example of low ‘Correlation’ value as a result of cellular phenotype, i.e., a low level of protein translocation into nuclei; green arrow – a field containing clumped cells that cannot be segmented properly; blue arrow – an example with both high ‘Correlation’ and *AR_cell_* values. In the latter, some artifacts were already excluded during image analysis through local illumination correction and constraints applied on the morphology and location of objects.


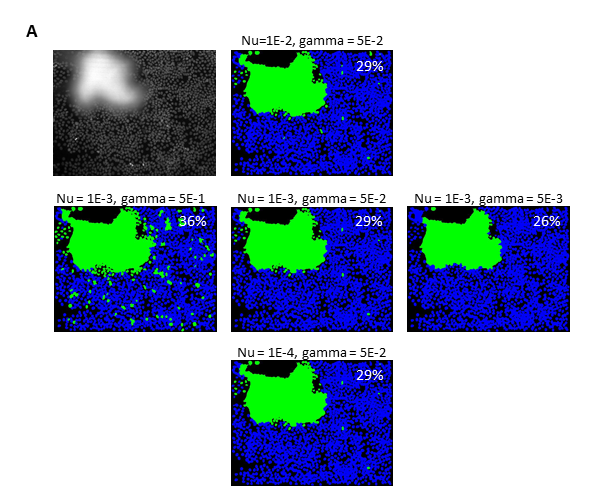


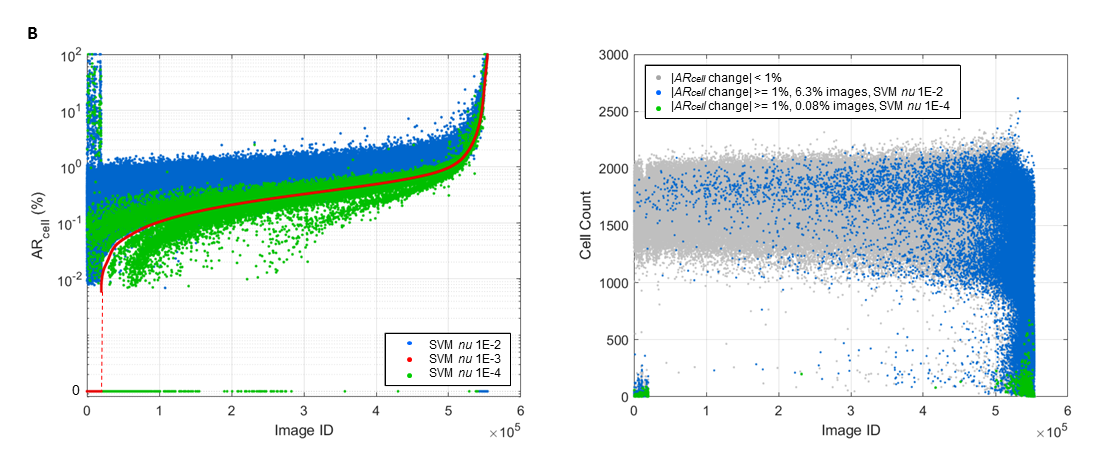


**Figure S5: Effect of one-class SVM hyperparameter *nu* and *gamma* variation on well image *AR_cell_* scores.** (A) An example image with its corresponding QC masks and *AR_cell_* scores under various combinations of *nu* and *gamma*. (B, left) Images are ordered along the x-axis by their *AR_cell_* score at our chosen value of *nu* = 1E-3 and plotted alongside the scores those images receive at *nu* =1E-2 and *nu* =1E-4, with *gamma* fixed at the default value (5E-2 for this assay). (B, right) Plot of the magnitudes of *AR_cell_* differences using the same ordering, with large magnitude differences shown in color by *nu* value. Images with lower than average cell counts display higher *AR_cell_* score sensitivity to either variation of *nu*, since changing the classification of a single cell has a greater effect on the artifact area ratio for that image.


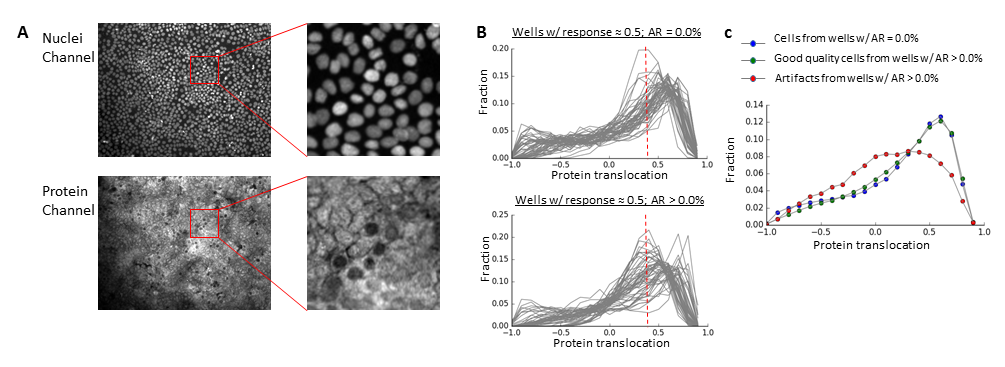


**Figure S6: Cell-level QC improves the accuracy of population analysis.** (A) A subpopulation of the cells have the protein of interest translocated into nuclei, demonstrating phenotypic heterogeneity. (B) Histogram of cellular phenotype for wells with response around 0.5 and *AR_cell_* equal to 0.0% (top) or greater than 0.0% (bottom). Each curve represents one well. The readout of protein translocation indicates a transition from zero to complete translocation into nucleus with its value changing from -1.0 to 1.0. Well-level response is characterized by the fraction of cells with this translocation measurement greater than 0.4 (red dashed line), a cutoff that achieves the largest separation window between positive and negative controls as measured by the Z’-factor. Compared to wells with ‘*AR_cell_* = 0.0%’, the distribution pattern of protein translocation is less consistent across wells with ‘*AR_cell_* > 0.0%’. (C) After removing the artifacts presenting an artificial distribution of protein translocation (red dots), the pattern of population heterogeneity was restored (green vs. blue dots). Histograms are made using cells collected from all wells with ‘response ≈ 0.5’.


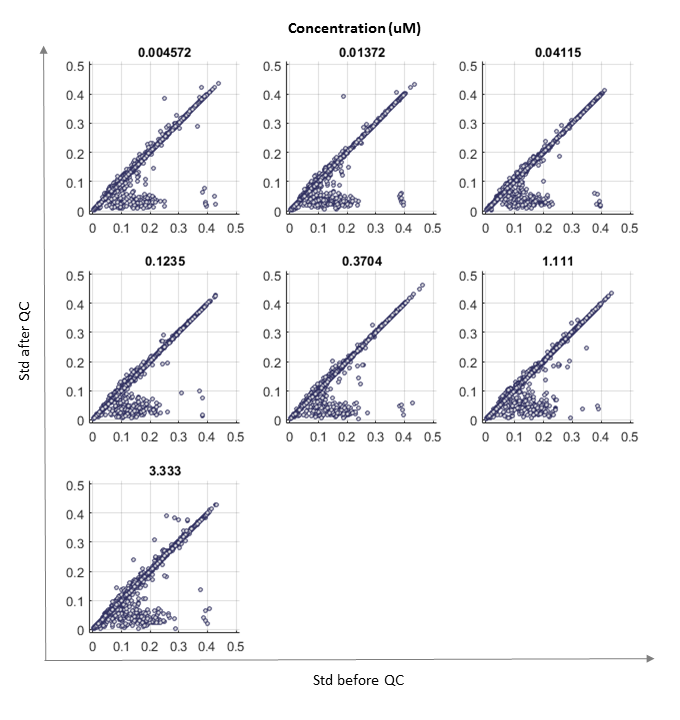


**Figure S7: Dose response consistency across replicates before and after cell-level QC, assay α.** To examine consistency, at each concentration, the standard deviation of drug response across six replicates was calculated and compared before and after cell-level QC. After removing artificial cells from individual wells, well-level response was recalculated. For this assay, wells with *AR_cell_* greater than 50% were disqualified from characterizing drug response and excluded before final statistics. Each circle represents one compound.


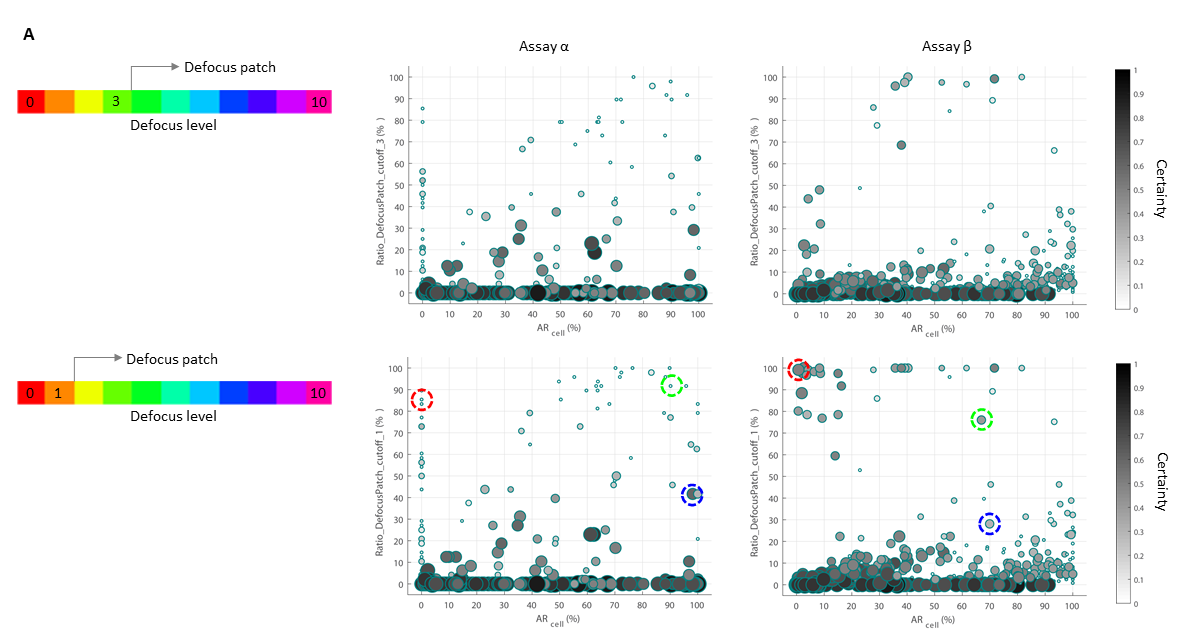


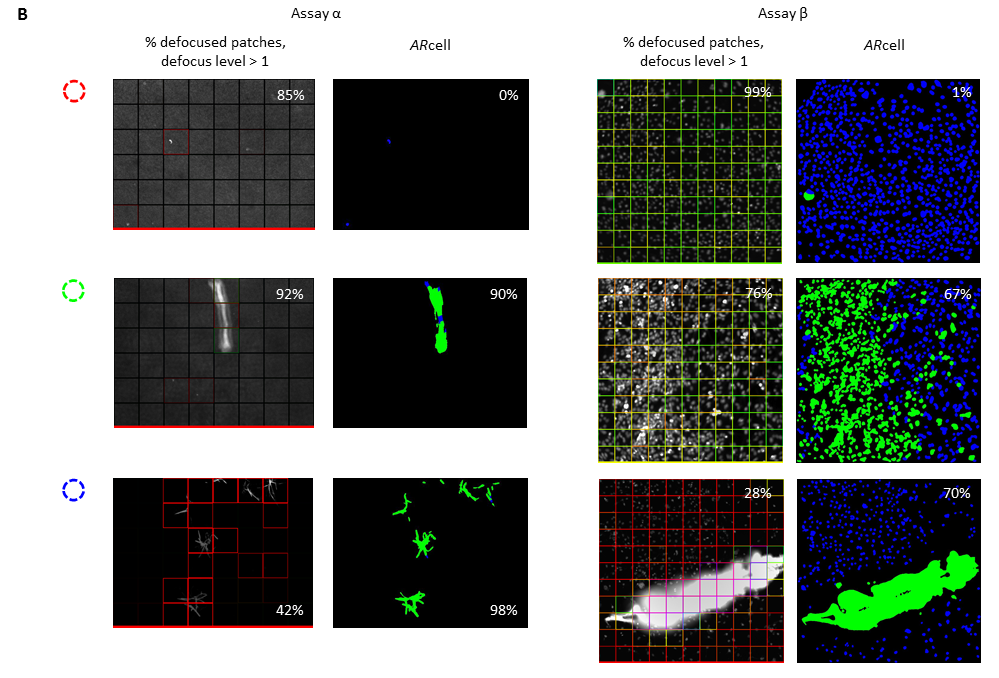


**Figure S8: A comparison between *AR_cell_* and defocused patch ratio under different defocus level cutoffs.** (A) The ratio of patches with defocus level greater than 3 (top) or 1 (bottom) is calculated for images from assay α and β and compared against *AR_cell_*. Each dot represents an image with both the size and intensity proportional to the prediction certainty of defocus level. Labeled patches and cell QC masks of examples from colored circles are given in (B).
